# Low-Level Cerebrospinal Fluid β-hCG Elevation in Patients with Pineal Parenchymal Tumors: A Four-Case Series and Diagnostic Caution

**DOI:** 10.3390/jcm15186934

**Published:** 2026-09-08

**Authors:** Yixuan He, Chuhong Tong, Yanong Li, Tao Jiang, Bo Li

**Affiliations:** 1 Department of Pediatrics, Beijing Tiantan Hospital, Capital Medical University, Beijing 100070, China; 2 Department of Radiation Oncology, Beijing Tiantan Hospital, Capital Medical University, Beijing 100070, China; 3 Department of Neurosurgery, Beijing Children’s Hospital, Capital Medical University, National Center for Children’s Health, Beijing 100045, China

**Keywords:** pineal parenchymal tumor, pineoblastoma, beta-human chorionic gonadotropin, intracranial germ cell tumor, germinoma, diagnostic caution

## Abstract

**Background**: Pineal region tumors (PRTs) are rare and biologically heterogeneous neoplasms with overlapping clinical and radiological features. Low-level cerebrospinal fluid (CSF) beta-human chorionic gonadotropin (β-hCG) may support a clinical diagnosis of germinoma (GE); however, its pathological specificity remains uncertain. **Methods**: We retrospectively reviewed four patients with low-level CSF β-hCG elevation whose available tissue specimens demonstrated pineal parenchymal tumors (PPTs). Tissue was obtained before systemic treatment in two patients and after platinum-based chemotherapy in two. **Results**: All four patients were male and aged 8 to 19 years. Pretreatment specimens from two patients demonstrated pineal parenchymal tumors of intermediate differentiation (PPTID), whereas postchemotherapy specimens from the other two demonstrated pineoblastoma (PB). Serum β-hCG was <0.1 IU/L in all patients, while CSF β-hCG ranged from 2.86 to 18.29 IU/L; serum and CSF alpha-fetoprotein (AFP) levels were normal. Two patients received platinum-based chemotherapy for presumptive intracranial germ cell tumors before tissue diagnosis, achieving stable disease or partial response. Because their specimens were obtained after chemotherapy, an occult pretreatment germ cell component could not be excluded. **Conclusions**: Although contemporary diagnostic protocols allow a clinical diagnosis of GE with characteristic imaging findings and low-level β-hCG elevation, isolated low-level CSF β-hCG immunoreactivity may also coexist with tissue specimens demonstrating PPT and should therefore not be considered pathognomonic for GE or interpreted in isolation. Early tissue acquisition with integrated histopathological and molecular evaluation is essential to avoid diagnostic misclassification and ensure appropriate treatment.

## 1. Introduction

Pineal region tumors (PRTs) are rare neoplasms of the central nervous system (CNS) representing under 1% of intracranial tumors in adults and roughly 3–8% of pediatric brain tumors. Their deep-seated anatomical location, overlapping radiological features, and heterogeneous histopathology make accurate pretreatment diagnosis difficult. According to the 2021 World Health Organization (WHO) Classification of Tumors of the CNS, the differential diagnosis of a pineal region mass includes intracranial germ cell tumors (iGCTs), pineal parenchymal tumors (PPTs), gliomas, meningiomas, and metastatic lesions [1,2,3]. IGCTs and PPTs predominantly affect children, adolescents, and young adults [4,5], frequently presenting with similar symptoms secondary to obstructive hydrocephalus or compression of adjacent structures, yet they differ substantially in therapeutic strategies and prognoses.

Measurement of alpha-fetoprotein (AFP) and the beta subunit of human chorionic gonadotropin (β-hCG) in serum and cerebrospinal fluid (CSF) is a key component of the diagnostic workup and subclassification of iGCTs [6,7,8]. In several contemporary diagnostic algorithms, protocols developed by several cooperative groups allow clinical diagnosis of germinoma (GE) without histopathological confirmation for carefully selected patients. For example, the COG protocols allow enrolment of patients with pineal or suprasellar tumors presenting with normal AFP levels and only mild elevation of serum/CSF β-hCG (5 to <50 IU/L). For SIOP protocols, clinical diagnosis is also acceptable for bifocal lesions (pineal + suprasellar) accompanied by diabetes insipidus in patients aged over 8–10 years [6]. Low-level CSF β-hCG immunoreactivity has been reported predominantly in iGCT, while occasional observations have also been described in patients with non-germ-cell tumors of the pineal region. Herein, we report four histopathologically confirmed PPT cases (two PPTIDs and two PBs), delineate this underappreciated clinical entity, highlight potential limitations in interpreting CSF β-hCG results, and emphasize the critical role of pathology-driven diagnosis for PRTs. This work also alerts clinicians to potential diagnostic caveats and provides actionable guidance for clinical management decisions.

## 2. Materials and Methods

We retrospectively reviewed patients treated at Beijing Tiantan Hospital, Capital Medical University between December 2023 and November 2025. The inclusion criteria were as follows: (a) a primary PRT; (b) serum or CSF β-hCG above the institutional upper reference limit, with normal serum and CSF AFP levels; and (c) a histopathological diagnosis by resection or biopsy of PPT. The exclusion criteria were as follows: (a) recurrent disease and (b) uncertain pathology, or incomplete clinical or imaging data.

Clinical, radiological, laboratory, histopathological, molecular, treatment, and follow-up data were systematically collected. β-hCG levels were measured using electrochemiluminescence immunoassays with the Elecsys HCG + β kit (Roche Diagnostics GmbH, Mannheim, Germany) on a cobas e601 analyzer in the hospital laboratory. The institutional reference range for β-hCG was 0–2.6 IU/L (functional sensitivity < 0.6 IU/L; limit of detection ≤ 0.1 IU/L), and the range for AFP was 0–7 ng/mL in our institute. CSF samples were obtained through lumbar puncture or ventricular CSF collected intra-operatively. Paired serum and CSF β-hCG and AFP values were recorded at initial diagnosis and during treatment when available. Isolated low-level CSF β-hCG elevation was defined as a CSF concentration above the institutional upper reference limit (2.6 IU/L) in the presence of normal serum β-hCG and normal serum and CSF AFP concentrations.

All patients underwent preoperative neuroimaging evaluation, including 3.0-T magnetic resonance imaging (MRI) and computed tomography (CT). Imaging findings were independently reviewed by two experienced neuroradiologists who were blinded to the pathological diagnosis. Histology, immunohistochemistry, Ki-67 proliferation index, and DNA methylation profiling were recorded when available. Tissue specimens were obtained before the initiation of systemic therapy in Cases 1 and 2, whereas Cases 3 and 4 underwent platinum-based chemotherapy before surgical resection. Consequently, the pathological diagnoses in Cases 3 and 4 represent the histological and molecular characteristics of the residual postchemotherapy tumors. Because pretreatment tissue was unavailable in these two patients, the presence of an occult chemotherapy-sensitive germ cell component in the original tumors could not be definitively excluded.

This study was approved by the Ethics Committee of Beijing Tiantan Hospital, Capital Medical University (KYSQ 2026-253-01). We have de-identified all patient details, ensuring confidentiality and anonymity of the data collected. The study was reported in accordance with the Case Report (CARE) guidelines [9].

## 3. Case Presentation

### 3.1. Case 1

**Figure 1:**
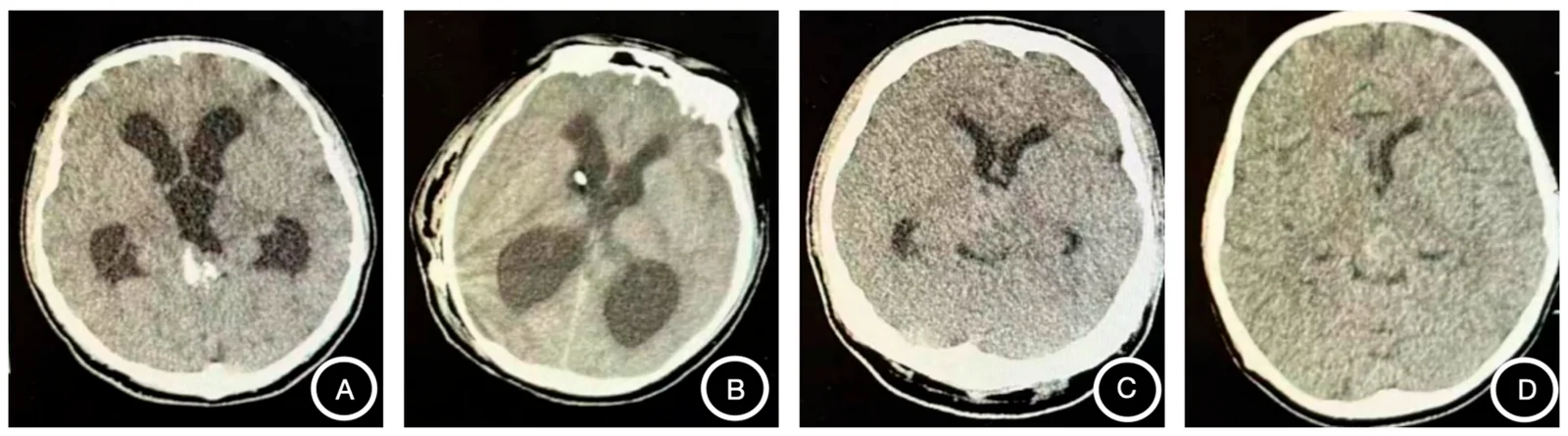
Axial computed tomography images. (**A**) Case 1, a 17-year-old male: irregular mixed-density mass in the posterior third ventricle/pineal region with multiple calcifications. (**B**) Case 2, a 15-year-old male: posterior third ventricular isodense mass with punctate and plaque-like calcifications. (**C**) Case 3, a 19-year-old male: irregular mildly hyperdense pineal region mass. (**D**) Case 4, an 8-year-old male: irregular mildly hyperdense pineal region mass with heterogeneous density and small hypodense foci.

**Figure 2:**
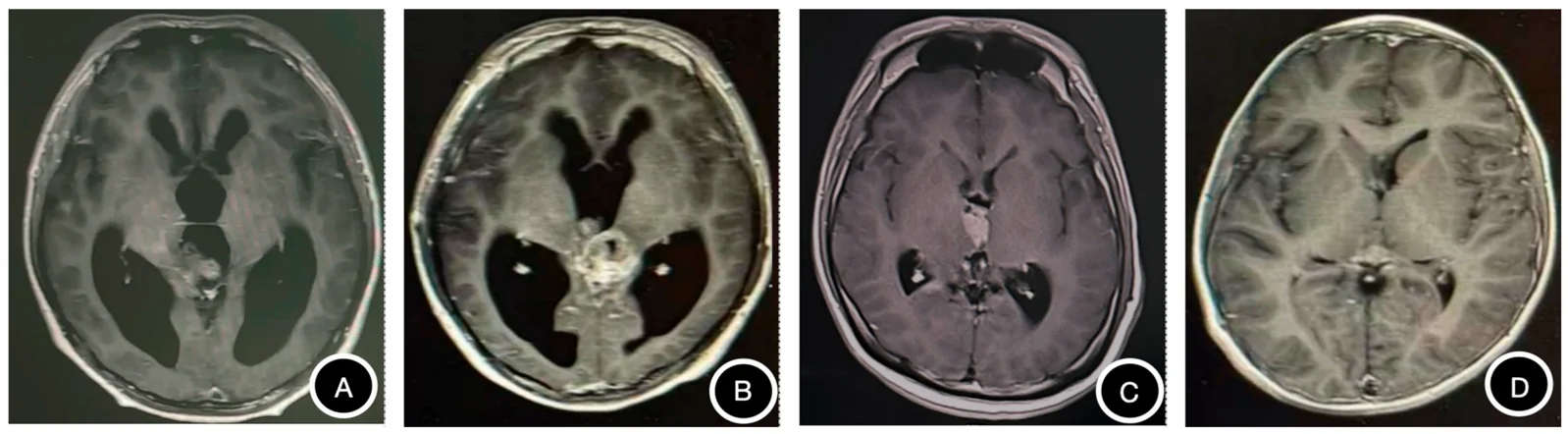
Contrast-enhanced axial T1-weighted magnetic resonance images. (**A**) Case 1: irregular cystic–solid mass in the posterior third ventricle/pineal region, with strong enhancement of the solid component and cyst wall. (**B**) Case 2: mixed-signal posterior third ventricular mass with heterogeneous nodular and patchy enhancement. (**C**) Case 3: cystic–solid third ventricular mass with marked heterogeneous enhancement. (**D**) Case 4: pineal region and third ventricular mass with mild-to-moderate heterogeneous enhancement.

A 17-year-old boy presented with a more than 2-year history of hand tremor. Neurological examination demonstrated bilateral upper-limb tremor without focal motor deficits. Laboratory examination showed an elevated CSF β-hCG level of 8.89 IU/L, whereas serum β-hCG was below 0.1 IU/L. Serum and CSF AFP levels were within the reference ranges. CT (Figure 1A) revealed a mixed-density pineal region mass (28 × 25 × 31 mm) containing multiple punctate and patchy calcifications. MRI (Figure 2A) revealed a heterogeneous mass (27 × 17 × 19 mm). The solid component exhibited iso- to hypointense signal on T1-weighted imaging, slightly hyperintense signal on T2-weighted and FLAIR imaging, and patchy enhancement. Obstructive hydrocephalus, periventricular edema, downward displacement of the third ventricle, and cerebellar tonsillar herniation were also observed. The initial radiological differential diagnoses included iGCT and PPT.

The patient underwent endoscopic third ventriculostomy and biopsy. Histopathological examination established a diagnosis of PPTID, CNS WHO grade 2. Immunohistochemical staining was positive for Syn, CgA, NeuN, S100, and GFAP. The Ki-67 labeling index was approximately 5–15%, and immunostaining for β-hCG and AFP were negative.

Subsequently, the tumor was surgically resected because the obstructive hydrocephalus did not resolve completely. CSF β-hCG remained mildly elevated at 4.47 IU/L after biopsy and 7.18 IU/L after resection. The patient subsequently received radiotherapy and remained alive without evidence of disease at the most recent follow-up.

### 3.2. Case 2

A 15-year-old boy was admitted due to a 2-week history of headache and a 4-day history of dizziness. The neurological examination revealed no abnormalities. CSF showed an elevated β-hCG level of 18.29 IU/L, whereas serum β-hCG remained below the detection limit and serum and CSF AFP levels were within the reference ranges. CT (Figure 1B) showed a mixed-density mass with multiple intratumoral calcifications. MRI (Figure 2B) showed an irregular cystic–solid mass (31 × 42 × 22 mm) involving the posterior third ventricular and pineal region, with hypointense signal on T1-weighted, hyperintense signal on T2-weighted and FLAIR imaging in the solid component, and marked enhancement of the solid component and cyst wall. Diffusion restriction and obstructive supratentorial hydrocephalus were present, raising suspicion for iGCTs.

The patient initially underwent ventriculoperitoneal shunting followed by biopsy. Histopathology demonstrated a PPTID, CNS WHO grade 2–3. Immunohistochemical staining was positive for Syn and NeuN, with a Ki-67 labeling index of approximately 15%. iGCT-associated markers, including OCT3/4, SALL4, AFP, and β-hCG, were negative.

The patient subsequently underwent near-total tumor resection through a supracerebellar approach combined with endoscopic third ventriculostomy, followed by local radiotherapy. Follow-up MRI showed a slight reduction in the residual lesion, and the patient remained alive without evidence of disease at the most recent follow-up.

### 3.3. Case 3

A 19-year-old boy presented with headache and dizziness lasting for one month. Serum β-hCG and AFP levels were within the reference ranges, whereas CSF β-hCG was mildly elevated at 5.28 IU/L. CT (Figure 1C) demonstrated a mildly hyperdense pineal region mass without calcification. MRI (Figure 2C) revealed a 14 × 23 × 19 mm mass involving the posterior third ventricle and pineal region. The lesion contained multiple small cystic components and showed marked diffusion restriction and prominent heterogeneous enhancement. The solid component was hypointense on T1-weighted imaging, iso- to hyperintense on T2-weighted imaging, and relatively hypointense on FLAIR imaging.

Based on the patient’s age, tumor location, imaging findings, and low-level CSF β-hCG elevation, an iGCT was initially suspected. The patient received four cycles of cisplatin, etoposide, and ifosfamide chemotherapy, resulting in a partial radiographic response. Because a substantial residual tumor remained and no pretreatment tissue diagnosis was available, near-total resection was subsequently performed.

Histopathological examination of the postchemotherapy specimen demonstrated PB, CNS WHO grade 4. Immunohistochemical staining was positive for Syn and CgA and negative for H3K27M and EZHIP. The Ki-67 labeling index reached approximately 70% in hotspot areas. Persistent postoperative hydrocephalus required endoscopic third ventriculostomy, septostomy, and subsequent lumbar cisternal drainage. Treatment was subsequently adjusted to a PB-directed regimen consisting of craniospinal irradiation and chemotherapy. The patient was alive without evidence of disease at the most recent follow-up. Because the surgical specimen was obtained only after four cycles of chemotherapy, the pathological findings characterize the residual post-treatment tumor. An occult chemotherapy-sensitive germ cell component present before treatment cannot be definitively excluded.

### 3.4. Case 4

An 8-year-old boy presented with headache and blurred vision lasting for one month. Serum β-hCG was <0.1 IU/L, whereas CSF β-hCG was marginally elevated at 2.86 IU/L. Serum and CSF AFP levels were within the reference ranges. CT (Figure 1D) demonstrated a 19 × 17 × 17 mm mildly hyperdense pineal region mass with heterogeneous density and no calcification. MRI (Figure 2D) revealed a 19 × 21 × 19 mm pineal region mass that was approximately isointense on T1-weighted, T2-weighted, and FLAIR imaging and showed mild-to-moderate heterogeneous enhancement. Additional irregular areas of enhancement were observed along the surface of the brainstem and the cervical and thoracic spinal cord. An iGCT with possible leptomeningeal dissemination was initially suspected.

The patient received two cycles of carboplatin and etoposide chemotherapy without an objective radiographic response. Complete tumor resection was subsequently performed to obtain a definitive diagnosis. Histopathological examination of the postchemotherapy specimen demonstrated PB, CNS WHO grade 4, miRNA pathway-altered subtype. Molecular analysis identified chromosomal alterations involving 1p, 10, 11, 14q, 16, and 19, as well as a PDGFRA p.P1027R variant. Immunohistochemical staining was positive for Syn, INI1, and BRG1, with focal expression of NeuN and NF and negative staining for SALL4. The Ki-67 labeling index was approximately 30%.

The patient received additional postoperative chemotherapy. At the latest follow-up, enhancement along the spinal cord surface remained largely unchanged, and the patient was alive with controlled disease while continuing treatment.

As no pretreatment tissue was available, the PB diagnosis reflects the composition of the residual tumor after chemotherapy. Although the absence of a meaningful radiographic response argues against a predominantly chemotherapy-sensitive germ cell tumor, it does not completely exclude a small germ cell component that may have been eradicated before resection.

CSF sampling was performed at different time points and in relation to different neurosurgical procedures according to the clinical conditions. Sampling site, timing relative to CSF diversion and tumor-directed procedures, and paired serum tumor-marker measurements were retrospectively reviewed and summarized in Supplementary Table S1.

## 4. Discussion

PRTs comprise a biologically heterogeneous group of neoplasms that frequently exhibit overlapping clinical manifestations and neuroimaging characteristics [10]. Given the substantial differences in therapeutic strategies and prognostic outcomes between GEs and PPTs, establishing an accurate diagnosis before treatment initiation is of critical importance. The primary aim of this study was to describe the diagnostic caution associated with isolated low-level CSF β-hCG elevation in patients whose available tissue specimens demonstrated PPT.

Serum and CSF AFP and β-hCG constitute important components of the diagnostic evaluation of iGCTs. Contemporary cooperative-group protocols have increasingly adopted risk-adapted diagnostic strategies that allow for a clinical diagnosis of iGCTs without histopathological confirmation in selected patients. In the COG ACNS2321 study, patients with typical pineal or suprasellar tumors, normal AFP, and serum/CSF β-hCG levels of 5 to <50 IU/L could be enrolled and treated as GE without histologic confirmation. Patients with bifocal (pineal and suprasellar) involvement or with diabetes insipidus in the setting of a pineal mass, provided serum/CSF β-hCG ≤ 100 IU/L and normal AFP levels, were also eligible. In the SIOP CNS GCT II study, a clinical diagnosis of GE may be considered in selected patients older than 8–10 years presenting with diabetes insipidus, typical radiological findings and bifocal involvement. Conversely, if AFP > 25 ng/mL or β-hCG > 50 IU/L, a clinical diagnosis of NGGCT should be considered. These approaches were developed to avoid unnecessary neurosurgical procedures in highly radiosensitive and chemosensitive tumors. Elevated AFP levels generally reflect the presence of yolk sac tumor differentiation, whereas markedly elevated β-hCG concentrations are typically associated with choriocarcinomatous or syncytiotrophoblastic differentiation [11,12,13]. However, despite their widespread clinical use, no universally accepted β-hCG threshold has been established to distinguish GE from NGGCT. CSF β-hCG is generally considered more sensitive than serum β-hCG [14]; however, greater sensitivity does not necessarily imply greater pathological specificity when a mildly elevated value is interpreted without considering the clinical, radiological, and histopathological context.

In this case series, we describe four patients with isolated low-level CSF β-hCG elevation whose available tissue specimens demonstrated PPT. Serum β-hCG was negative and serum and CSF AFP levels were within the reference ranges in all patients, whereas CSF β-hCG concentrations ranged from 2.86 to 18.29 IU/L (Table 1). The strength of the pathological evidence, however, differed among the four cases. Cases 1 and 2 underwent biopsy before systemic treatment, providing pretreatment histological evidence of PPTID in the setting of isolated low-level CSF β-hCG elevation. By contrast, Cases 3 and 4 received platinum-based chemotherapy before tissue acquisition, and the subsequent resection specimens demonstrated PB. These latter findings confirm the presence of PB in the residual tumors but cannot establish the complete histological composition of the tumors before chemotherapy.

The diagnostic challenges highlighted by these cases reflect a multifactorial process rather than a single interpretive error. The overlap between GEs and PPTs in demographic characteristics, presenting symptoms, and imaging features creates a substantial diagnostic challenge. Both entities predominantly affect children, adolescents, and young adults and frequently present with symptoms related to obstructive hydrocephalus, including headache, nausea, visual impairment, and gait disturbance. Moreover, conventional CT and MRI findings often demonstrate considerable overlap, particularly among lesions with mixed solid–cystic architecture, heterogeneous enhancement patterns, and variable calcification [15,16,17]. Consequently, imaging alone may not provide sufficient diagnostic confidence in some cases. Endoscopic biopsy provides a practical diagnostic option for patients presenting with obstructive hydrocephalus, as it allows pathological sampling while simultaneously providing CSF diversion through endoscopic third ventriculostomy. Immunohistochemical profiling and molecular classification can further support accurate CNS tumor classification and treatment selection [17].

Within this context, particularly when surgical accessibility is considered technically challenging, even a low-level elevation of CSF β-hCG may exert a disproportionate influence on clinical decision-making. Our experience suggests that reliance on tumor-marker interpretation alone may result in premature initiation of treatment before pathological confirmation. Such an approach not only carries the risk of ineffective treatment exposure but may also delay definitive management.

The diagnostic significance of low-level CSF β-hCG elevation remains a matter of ongoing debate [18,19,20,21]. Allen et al. reported that approximately one-third of histologically confirmed GEs showed elevated CSF β-hCG despite normal serum levels, supporting the greater sensitivity of CSF analysis for detecting β-hCG within the central nervous system [22]. Hu et al. proposed a CSF β-hCG cutoff value of 8.2 IU/L, which improved diagnostic sensitivity for iGCT compared with serum measurements [14]. Although CSF β-hCG is generally more sensitive than serum β-hCG for detecting iGCTs, higher sensitivity does not necessarily translate into greater diagnostic specificity [23]. Importantly, these studies mainly focused on patients with GE and included relatively limited control groups.

These findings should be interpreted differently according to the timing of tissue acquisition. Pretreatment PPTID was demonstrated in Cases 1 and 2, whereas the PB specimens in Cases 3 and 4 represented residual tumors after chemotherapy. Thus, the former support the coexistence of low-level CSF β-hCG elevation and PPTID before systemic treatment, while the latter cannot exclude an occult pretreatment germ-cell component. Treatment response cannot retrospectively determine the complete pretreatment histology: a partial or absent radiographic response does not exclude the possibility that a small chemotherapy-sensitive germ cell component was eliminated while a chemotherapy-resistant PB component persisted. Accordingly, an unexpected treatment response should prompt diagnostic reassessment and consideration of tissue diagnosis rather than continuation of an unconfirmed treatment strategy.

Our observations are consistent with the previous report by Bouille et al. describing PB in patients with isolated CSF β-hCG elevation [24]. In the present series, Cases 1 and 2 additionally demonstrated PPTID in pretreatment tissue. Three of the four patients had no evidence of metastatic disease; however, the small selected case series does not allow conclusions regarding the relationship between CSF β-hCG elevation and tumor dissemination.

In Case 1, serial CSF β-hCG measurements were obtained at different stages of neurosurgical management and from different CSF compartments, as detailed in Supplementary Table S1. Although the measured values did not parallel the apparent changes during surgical intervention, the sampling conditions were not directly comparable. Therefore, these serial measurements should be regarded as descriptive observations, and no conclusion regarding the monitoring performance of CSF β-hCG can be drawn from this single case.

All patients in our cohort exhibited low-level CSF β-hCG elevation with simultaneously negative serum β-hCG. The biological origin of the low-level CSF β-hCG measurements in these patients remains undetermined. Several non-mutually exclusive factors may contribute to these observations, including analytical and preanalytical variability, differences in CSF sampling compartments, and pathological sampling limitations. Different immunoassays detect different forms of hCG, including intact hCG, total hCG, and free β-subunit, and their performance at very low concentrations may vary considerably [25]. The β-subunit of hCG is encoded by the chorionic gonadotropin subunit beta (CGB) gene family; therefore, demonstration of CGB expression would provide more direct evidence of β-hCG production by tumor cells. Although focal ectopic β-hCG expression by PPT cells remains a theoretical possibility [2,10,26], no direct molecular evidence of CGB expression was available in the present study. Importantly, CSF β-hCG measurements were performed using a single clinical diagnostic electrochemiluminescence platform, and repeat testing on an independent analytical platform was not feasible due to lack of stored CSF aliquots. Therefore, our findings only demonstrate the coexistence of low-level CSF β-hCG elevation and histologically confirmed PPT specimens, and we cannot establish whether the measured β-hCG was derived from tumor cells, pre-analytical artifacts, or other biological sources.

Emerging CSF biomarkers, including placental alkaline phosphatase (PLAP), OCT3/4, circulating tumor DNA (ctDNA), and microRNA profiles, may further improve diagnostic assessment in the future [27,28,29,30,31,32]. However, current evidence remains insufficient, and these markers should be considered complementary tools rather than replacements for histopathological confirmation when tissue diagnosis is indicated. Prospective multicenter studies with standardized protocols are needed before these biomarkers can be incorporated into routine clinical decision-making.

A strength of this case series is the detailed clinicoradiological and pathological characterization of four diagnostically challenging PRTs. Pretreatment pathological evidence was available in Cases 1 and 2, while the postchemotherapy nature of the specimens in Cases 3 and 4 was explicitly considered in interpreting the findings. The reconstruction of tumor-marker measurements, CSF sampling sites, and major clinical interventions further allows the biochemical findings to be interpreted within their clinical context.

Several limitations should be acknowledged. First, this was a retrospective, single-center case series involving only four selected patients without a denominator or comparison group; therefore, the prevalence and diagnostic performance of low-level CSF β-hCG in PPTs cannot be determined. Second, the biological origin of the detected β-hCG remains uncertain, and direct molecular evidence of CGB expression was not available. Third, pretreatment tissue was unavailable in Cases 3 and 4, precluding complete exclusion of an occult chemotherapy-sensitive germ-cell component in the original tumors. Fourth, β-hCG levels were measured as part of routine clinical care, and low-level results were not systematically confirmed using an independent analytical platform. CSF-specific assay validation data were lacking, and variation in CSF sampling sites and timing limited interpretation of serial measurements. Follow-up duration also varied among patients. Future multicenter studies incorporating standardized biochemical testing, comprehensive pathological and molecular characterization, and long-term follow-up are needed to clarify these findings.

## 5. Conclusions

In summary, our findings highlight that low-level CSF β-hCG immunoreactivity can coexist with histologically diagnosed PPTs and therefore should not be considered pathognomonic for GE. In diagnostically discordant cases or when marker elevations are marginal, analytical confirmation and tissue diagnosis with integrated histopathological and molecular evaluation should be considered when clinically safe and feasible.

## Figures and Tables

**Table 1:** Clinical characteristics of four patients with PPT and low-level CSF β-hCG elevation.

Case	Age/Sex	Presentation/Stage	Serum Markers (β-hCG, IU/L; AFP, ng/mL)	CSF Markers (β-hCG, IU/L; AFP, ng/mL)	Initial Diagnosis	Pathology	Ki-67/Methylation Classification	Treatment	Outcome
1	17/M	Tremor/M0	β-hCG < 0.1; AFP 1.64	β-hCG 8.89; AFP < 0.605	Undetermined	PPTID, CNS WHO grade 2	5–15%; PPT; MGMT promoter unmethylation	ETV + biopsy → resection → RT	Alive, disease-free
2	15/M	Headache, dizziness/M0	β-hCG < 0.1; AFP 1.68	β-hCG 18.29; AFP < 0.605	Undetermined	PPTID, CNS WHO grade 2–3	15%; PPT; MGMT promoter unmethylation	VPS → biopsy → resection + ETV → RT	Alive, disease-free
3	19/M	Headache, dizziness/M0	β-hCG < 0.1; AFP 0.848	β-hCG 5.28; AFP < 0.605	iGCT	PB, CNS WHO grade 4, identified in a postchemotherapy specimen	70%; PB; methylation indeterminate	4 cycles ICE → PR → resection → RT	Alive, disease controlled
4	8/M	Headache, visual decline/M+	β-hCG < 0.1; AFP 1.08	β-hCG 2.86; AFP < 0.605	iGCT	PB, CNS WHO grade 4, miRNA pathway-altered, identified in a postchemotherapy specimen	30%; PB, miRNA-pathway alteration	VPS → 2 cycles CE → SD → resection → RT	Alive, disease controlled

AFP, alpha-fetoprotein; β-hCG, beta subunit of human chorionic gonadotropin; CE, carboplatin plus etoposide; ETV, endoscopic third ventriculostomy; ICE, cisplatin plus etoposide plus ifosfamide; iGCT, intracranial germ cell tumor; M0, no dissemination; M+, leptomeningeal dissemination; MGMT, O6-methylguanine-DNA methyltransferase; PB, pineoblastoma; PPT, pineal parenchymal tomor; PPTID, pineal parenchymal tumor of intermediate differentiation; PR, partial response; RT, radiotherapy; SD, stable disease; VPS, ventriculoperitoneal shunt.

## Data Availability

This is a descriptive case series. Anonymized data may be provided upon reasonable request.

## Supplementary Materials

The following supporting information can be downloaded at: https://www.mdpi.com/article/10.3390/jcm15186934/s1, Table S1: CSF and Serum β-hCG/AFP Monitoring Across Diagnostic and Neurosurgical Events.

## Abbreviations

The following abbreviations are used in this manuscript:

CSF	cerebrospinal fluid
β-hCG	beta-human chorionic gonadotropin
iGCT	intracranial germ cell tumor
PPTID	pineal parenchymal tumor of intermediate differentiation
PB	pineoblastoma
AFP	alpha-fetoprotein
PRT	pineal region tumor
CNS	central nervous system
WHO	World Health Organization
MRI	magnetic resonance imaging
CT	computed tomography
Syn	synaptophysin
CgA	chromogranin A
NeuN	neuronal nuclear antigen
GFAP	glial fibrillary acidic protein
CE	carboplatin plus etoposide
ETV	endoscopic third ventriculostomy
ICE	cisplatin plus etoposide plus ifosfamide
PR	partial response
SD	stable disease
VPS	ventriculoperitoneal shunt
RT	radiotherapy
MGMT	O^6^-methylguanine-DNA methyltransferase
COG	Children’s Oncology Group
NGGCT	non-germinomatous germ cell tumor
CGB	chorionic gonadotropin subunit beta
